# Design and Research of Intelligent Bearing Magnetic Coupling Piezoelectric–Triboelectric Composite Rotary Energy Harvester

**DOI:** 10.3390/s26092778

**Published:** 2026-04-29

**Authors:** Heng Wang, Wanru Sun, Zifei Li, Liucheng Zhu, Yuxuan Zhu, Haocheng Wang

**Affiliations:** 1 School of Mechanical Engineering, Nantong University, Nantong 226019, China; swr9523@outlook.com (W.S.); 2310310023@stmail.ntu.edu.cn (Z.L.); 2510310015@stmail.ntu.edu.cn (L.Z.); 2 Faculty of Science and Technology, Electromechanical Engineering, University of Macau, Macau 999078, China; dc32707@umac.mo; 3 Equipment Management and Unmanned Aerial Vehicle Engineering School, Air Force Engineering University, Xi’an 710051, China; 13951317560@163.com

**Keywords:** intelligent bearings, magnetic coupling, piezoelectric–triboelectric composite, rotational motion energy harvesting, self-powered

## Abstract

**Highlights:**

**What are the main findings?**

- A hybrid piezoelectric–triboelectric energy harvester was designed, achieving synergistic power generation from the piezoelectric and triboelectric units.
- Periodic vibrations generated by rotating magnetic forces serve as the vibrational excitation for a hybrid energy harvester, inducing a voltage output from the device.

**What are the implications of the main findings?**

- The designed hybrid energy harvester can be effectively utilized for the self-powering of smart bearings.

**Abstract:**

To address the issue of insufficient output voltage of the self-powered unit of intelligent bearings under low-amplitude working conditions, a piezoelectric–friction composite energy harvester driven by rotating magnetic force is proposed based on the multi-physical field coupling and synergy of magnetoelectric, piezoelectric and triboelectric effects, which effectively enhances the voltage output in low-amplitude vibration environments. The intelligent bearing adopts an extended structure, consisting of an outer ring sleeve, an inner ring extension ring, magnetic poles and a composite energy harvester. The outer ring sleeve is nested on the outer ring of the bearing and fixes the composite energy harvester, while the inner ring extension ring is fixed on the inner ring of the bearing and installs the magnetic poles. The composite energy harvester adopts a magnetic double-mass block single-crystal piezoelectric simply supported beam structure and integrates a contact-separation type triboelectric nanogenerator in the vibration direction, achieving the collaborative power supply of the piezoelectric and triboelectric units. A mechanical-electrical coupling dynamic model of the composite energy harvester is developed. Using COMSOL software, the effects of various structural dimensions and magnetic pole configurations on the output voltage are analyzed. Experimental validation confirms the model’s effectiveness. The results demonstrate that the energy harvester operates effectively under varying bearing rotational speeds. The rotational speed of the magnetic poles has little influence on the output voltage amplitude but primarily affects its frequency. Under the condition that the rotational speed is within 600 r/min, the piezoelectric module stably outputs a peak voltage of approximately 16.6 V, and the triboelectric unit stably outputs a peak voltage of approximately 4.4 V, which can effectively meet the self-driving requirements of intelligent bearings.

## 1. Introduction

Rolling bearings play an indispensable role in industrial development. As a critical mechanical component, they are widely used in various types of machinery and equipment. Typically, the operational status signals of bearings are obtained by installing vibration sensors on the bearing housing or bearing box. However, the signals collected in this manner not only contain information about the bearing’s operational status but also include noise signals generated by the movement of other mechanisms, which is highly detrimental to bearing fault monitoring [1]. To address this issue, the concept of smart bearings has been introduced, starting from the root of signal acquisition. Smart bearings consist of an improved bearing body, related accessories, microsensors, and processing/transmission circuits, forming a bearing unit with self-perception, self-diagnosis, and self-regulation capabilities [2,3]. This enables effective monitoring of the bearing’s operational status.

Currently, there are two main methods for connecting sensors to smart bearings: the external-attachment type and the embedded type. The embedded structure of smart bearings involves machining grooves on the bearing to accommodate sensors, allowing them to better capture relevant signals transmitted by the bearing. However, this compromises the structural integrity of the bearing and negatively impacts its operational performance. The external-attachment structure refers to sensors being integrated with the smart bearing through external components, preserving the bearing’s structural integrity while minimizing its impact on performance. This design also facilitates easier sensor installation. Robert X. Gao [4,5] first proposed the embedded smart bearing structure, where the sensing system is embedded into the bearing’s outer ring to capture more accurate operational signals. Shen Shaowei et al. [6] analyzed the stress conditions of rolling bearings under different rotational speeds and groove configurations, laying a foundation for research on groove dimensions in embedded smart bearings. While the embedded structure enhances signal reception, it still compromises structural integrity and affects performance. Germany’s FAG company developed an external-attachment deep groove ball bearing with a sensor system [7], which detects the relative motion between the inner and outer rings to obtain bearing operational signals. Sweden’s SKF company [8,9] introduced an external-attachment smart bearing capable of collecting both temperature and vibration acceleration signals. In addition to signal acquisition, it includes an energy harvesting system to power the sensors and utilizes cloud technology to enable information exchange among multiple bearings.

Due to the impact of the environment and the limited fossil fuels, the energy transition is very important, and the power supply of smart bearings requires new compact and efficient energy sources, which must address the limitations associated with conventional external power supplies, such as batteries with limited life and regular replacement. Currently, the main energy harvesting methods for most smart bearings are wired, battery, and wireless charging. Wired power supply involves complex cable connections, which are highly inconvenient in high-speed rotating bearing systems. The battery-powered approach requires mechanical shutdowns for battery replacement, leading to unnecessary cost losses. Wireless power supply necessitates external power coils, which are impractical for actual use in industrial settings. Therefore, self-powered technology plays a crucial role in the research of smart bearings. Currently, the more mature mechanical energy harvesting methods include electromagnetic [10,11,12], piezoelectric [13,14,15,16], and triboelectric nanogenerators [17,18,19].

Self-powered technology is fundamental to smart bearings, and many scholars worldwide have conducted research on vibration energy harvesting for bearings. As an excellent electromechanical transduction material, piezoelectric materials offer advantages such as compact size, low cost, and reliable performance. Qin Chaoye et al. [20] proposed a piezoelectric energy harvester that places curved piezoelectric elements between the bearing outer ring and housing to capture the motion energy of rotating machinery. However, this structure requires a new bearing support system and imposes certain environmental constraints. Huang Manjuan et al. [21] introduced a miniature piezoelectric energy harvesting device based on an impact-based frequency-up conversion mechanism, which primarily relies on the resonance effect of a cantilever beam. This device converts low-frequency environmental vibrations into high-frequency vibrations of the piezoelectric beam, effectively generating piezoelectric voltage. However, the installation of the resonant beam demands specific spatial requirements, making it impractical for real-world applications. Among various mechanical energy harvesting technologies, electromagnetic generators are the most widely used and technically mature. Cao Junyi et al. [22] developed a ring-shaped Halbach electromagnetic energy harvester designed for self-powered smart bearings, which harvests electrical energy from bearing rotation to power monitoring units. However, electromagnetic harvesters typically require significant installation space and fixed rotating components, which are difficult to accommodate within the compact structure of bearings. Cui Baozhen et al. [23] integrated an electromagnetic structure with the bearing to form a unified power supply system. Nevertheless, the low voltage output of electromagnetic structures limits their compatibility with certain sensors. Triboelectric nanogenerators (TENGs) generate output voltage by coupling the triboelectric effect with electrostatic induction. Zhao Xuejun [24] proposed a TENG-based energy harvesting device for train bogies, primarily capturing low-frequency vibrations to intermittently power the monitoring and data transmission systems of train wheel bearings. However, this design is limited to train bearings and has restricted applicability. Gao Shuai et al. [25] proposed an ultra-high-speed hybrid ceramic rolling-element triboelectric bearing, which enabled the real-time monitoring of bearing dynamic instability; however, due to the use of custom-tailored materials, this design faces certain limitations in practical applications. Han Qinkai et al. [26] introduced a wave-shaped cage-based triboelectric rolling bearing for fault diagnosis in rotating machinery, but its overly compact structure is prone to damage during operation. Hybrid energy harvesting technologies have also been explored. Choi et al. [27] designed an inexpensive triboelectric–piezoelectric sensor using a roller-bearing configuration of rollers and electrodes. The smart bearing outputs electrical signals upon rotation or displacement of the attached object. Xi Yi et al. [28] developed a contact-separation hybrid triboelectric nanogenerator based on rolling friction, combined with an electromagnetic generator (magnet-copper coil) inside a rolling cylinder, forming a hybrid energy harvesting system for bearing rotation. However, this design significantly alters the bearing structure, limiting its use to specific scenarios. Luo Mengting et al. [29] designed a smart bearing energy harvester based on a 6220 deep groove ball bearing to collect vibration energy during operation. However, the embedded structure compromises bearing integrity, and the low vibration amplitude results in insufficient output voltage, restricting its application. The aforementioned studies represent valuable attempts in self-powered rolling bearing technology, yet they share certain shortcomings: most devices modify the internal structure or materials of bearings, potentially affecting normal operation; electromagnetic structures produce low output voltage, limiting their applicability; and composite vibration energy harvesters suffer from output voltage instability due to vibration amplitude, possibly failing to meet the self-powering demands of smart bearings.

In this paper, a piezoelectric–triboelectric composite energy harvester with a rotating magnetic drive is proposed, which adopts an external extension structure to combine with the intelligent bearing through the external structure, which retains the integrity of the bearing structure, has little impact on the working performance of the bearing, and is convenient for sensor installation. Compared with the existing energy harvesting device, the magnetic coupling rotary energy harvester does not change the structure and material of the bearing itself, the periodic vibration amplitude provided by the rotating magnetic force is stable, and the triboelectric and piezoelectric structures have the advantages of high output voltage, and the two can make more efficient use of the energy generated by vibration, which is of great significance to the design of intelligent bearings.

## 2. Structural Design and Modeling of a Smart Bearing Rotational Energy Harvester

### 2.1. Overall Structural Design and Working Principle

**Figure 1:**
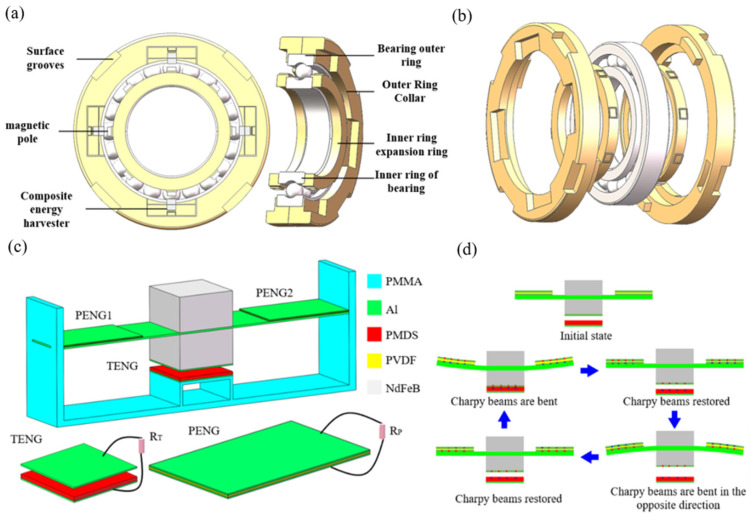
Design structure and functional principle of the energy harvester: (**a**) The overall structure of energy capture of intelligent bearings; (**b**) bearing fit configuration diagram; (**c**) structure diagram of the rotating energy harvester; (**d**) basic working concept of the composite energy harvester.

According to the current Z1 standard for rolling bearing vibration acceleration measurement [30], the acceleration amplitude of a 6220 deep groove ball bearing during normal operation ranges between 0 and 28.2 m/s^2^. Under different working conditions, the vibration amplitude generated by bearing operation varies significantly, resulting in considerable fluctuation in the excitation received by the energy harvester and consequently unstable output voltage, particularly with low-amplitude vibrations producing insufficient voltage. In contrast, the periodic vibrations generated by rotating magnetic forces demonstrate stable and controllable amplitudes, making them ideal excitation sources for energy harvesters while preserving the bearing’s structural integrity. As shown in Figure 1a, the overall structure of the smart bearing energy harvesting system adopts an external expansion design, consisting of a 6220 deep groove ball bearing, an outer ring sleeve, an inner ring extension, magnetic poles, and a hybrid energy harvester. The outer ring sleeve is fixed to the bearing’s outer race and features four grooves on one side to secure the hybrid energy harvester. The simply supported beam of the harvester is equipped with an NdFeB magnet on its upper side and a non-magnetized NdFeB material recess on the lower side. The groove dimensions are determined by the harvester size and the spacing between magnetic poles, with surface grooves designed to accommodate sensors and wireless components. The inner ring extension is mounted on the outer side of the bearing’s inner race and incorporates groove structures to fix NdFeB magnets. Both the outer sleeve and inner extension employ interference fit installation with their respective bearing rings, forming integrated assembly structures as illustrated in Figure 1b. The geometric parameters and materials of the overall structure are summarized in Table 1.

The composite energy harvester structure, as shown in Figure 1c, is primarily composed of a polymethyl methacrylate (PMMA) support base, one triboelectric generator unit (TENG), and two piezoelectric generator units (PENG1 and PENG2). A simply supported beam is fixed between the left and right sides of the support base, with an NdFeB magnet mounted on its upper center portion and non-magnetized NdFeB alloy material positioned below, forming an integrated mass block for the piezoelectric structure. The beam is flanked by two piezoelectric units, each featuring polyvinylidene fluoride (PVDF) as the piezoelectric material sandwiched between aluminum (Al) electrodes. The lower mass block incorporates an Al friction electrode, which interacts with a polydimethylsiloxane (PDMS) friction film backed by an Al electrode that is fixed to the central rear portion of the support base, collectively constituting the triboelectric generator unit. During bearing operation, while the energy harvester assembly remains stationary on the outer ring sleeve, the magnetic poles mounted on the inner ring extension rotate synchronously with the inner race. This configuration creates a dynamic magnetic interaction between the stationary poles on the harvester and the rotating poles on the inner ring. Under this periodic magnetic excitation, the composite energy harvester undergoes controlled vibrational motion. As illustrated in Figure 1d, the operational mechanism proceeds through distinct phases: In the initial repulsive phase, magnetic repulsion forces cause downward deflection of the simply supported beam structure. This deformation bends the PVDF-based piezoelectric units (PENG1/PENG2), generating the first polarity of piezoelectric output. Simultaneously, the aluminum friction electrode, integrated with the inertial mass block, makes contact with the PDMS triboelectric layer, resulting in electron transfer from the Al electrode to the PDMS surface and establishing opposite surface charges. During the subsequent attractive phase, magnetic attraction forces reverse the beam’s motion. The PVDF elements return toward their neutral position and undergo reverse bending, producing inverted polarity piezoelectric output. The separation between the Al electrode and PDMS film creates an increasing gap, establishing a measurable potential difference that generates the triboelectric signal.

### 2.2. Dynamic Model

**Figure 2:**
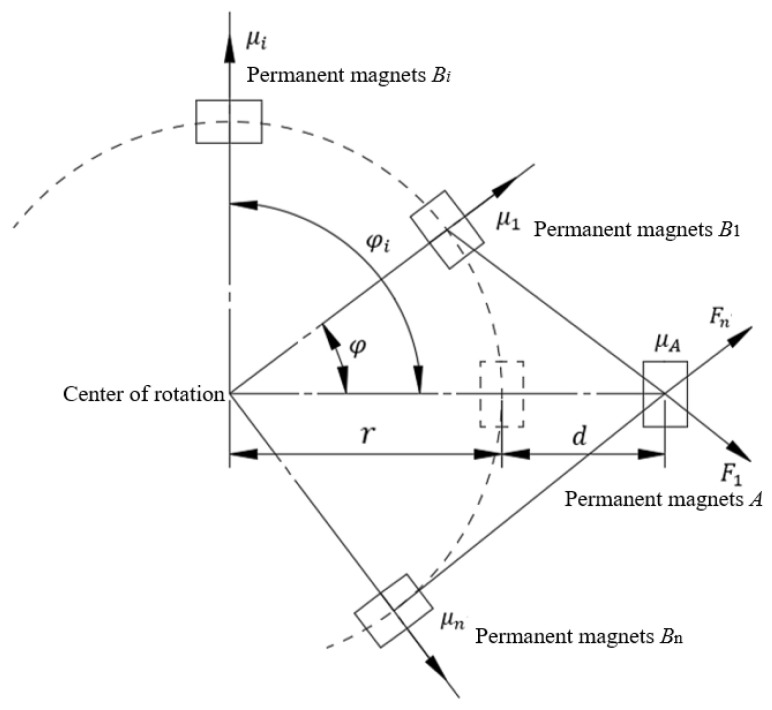
Motion and force analysis of the magnetic module.

To analyze the dynamic characteristics of the rotationally magnetically driven energy harvester, a magnetic coupling dynamic model for rotational motion was established based on the Lagrange equation. Figure 2 illustrates the motion and force conditions of the magnetically coupled rotational energy harvester. By utilizing the magnetic field superposition effect of multiple excitation magnetic poles, the total magnetic force acting on the harvester’s magnet can be determined. The kinetic energy of the rotating permanent magnet is expressed as:

(1) $$T=\frac{1}{2}nmr^{2}{\dot{\varphi }}^{2}$$

where *n* is the number of rotating poles, m is the mass of the magnetic pole, *r* is the radius of rotation of the magnetic pole, and *φ* is the angular displacement.

The magnetic moment vector *A* of the permanent magnet fixed on the energy harvester can be written as:

(2) $$\mu_{A}=-M_{A}V_{A}{\hat{e}}_{x}$$

where *M_A_* is the magnetization vector magnitude; *V_A_* is the volume of the permanent magnet; $M_{A}=B_{r}/\mu_{0}$; $B_{r}$ is the magnetic induction intensity of the permanent magnet; $\mu_{0}$ is the permeability of free space, and the magnetic moment vector $\mu_{i}$ of the rotating magnet $B_{i}$ is:

(3) $$\mu_{i}={\left(-1\right)}^{\left(i-1\right)}(M_{B}V_{B}\cos\varphi_{i}{\hat{e}}_{x}+M_{B}V_{B}\sin\varphi_{i}{\hat{e}}_{y})$$

The distance from $\mu_{i}$ to $\mu_{A}$ $r_{i}$ can be expressed as:

(4) $$r_{i}=-(r-r\cos\varphi_{i}+d+\delta )e_{x}+r\sin\varphi_{i}e_{y}$$

where d is the minimum center-to-center distance between the rotating and fixed magnets. δ is the deformation of the energy harvester, then the magnetic field generated by the fixed magnet A on the rotating magnet $B_{i}$ is:

(5) $$B_{i}=-\frac{\mu_{0}}{4\pi }\nabla \frac{\mu_{A}}{{\left\|r_{i}\right\|}_{2}^{3}}$$

The total potential energy stored in the magnetic field is:

(6) $$U_{mi}=-B_{i}•\mu_{i}$$

Deriving the potential energy of the magnetic field in the horizontal direction, the component of the magnetic force in the horizontal direction and the component of the magnetic repulsion force in the direction can be obtained:

(7) $$\begin{array}{l} F_{i}=M_{m}\left\{\frac{r\sin^{2}\varphi_{i}-3\left(r-r\cos\varphi_{i}+d\right)\cos\varphi_{i}}{{\left[{\left(r-r\cos\varphi_{i}+d\right)}^{2}+r^{2}\sin^{2}\varphi_{i}\right]}^{5/2}}-\frac{5{\left(r-r\cos\varphi_{i}+d\right)}^{2}\left[r\sin^{2}\varphi_{i}-3\left(r-r\cos\varphi_{i}+d\right)\cos\varphi_{i}\right]}{{\left[{\left(r-r\cos\varphi_{i}+d\right)}^{2}+r^{2}\sin^{2}\varphi_{i}\right]}^{7/2}}\right\} \end{array}$$

Component of the magnetic force of attraction in the direction:

(8) $$F_{i}=-M_{m}\left\{\frac{r\sin^{2}\varphi_{i}-3\left(r-r\cos\varphi_{i}+d\right)\cos\varphi_{i}}{{\left[{\left(r-r\cos\varphi_{i}+d\right)}^{2}+r^{2}\sin^{2}\varphi_{i}\right]}^{5/2}}-\frac{5{\left(r-r\cos\varphi_{i}+d\right)}^{2}\left[r\sin^{2}\varphi_{i}-3\left(r-r\cos\varphi_{i}+d\right)\cos\varphi_{i}\right]}{{\left[{\left(r-r\cos\varphi_{i}+d\right)}^{2}+r^{2}\sin^{2}\varphi_{i}\right]}^{7/2}}\right\}$$

where $M_{m}=3M_{A}V_{A}M_{B}V_{B}\mu_{0}/(4\pi )$.

The concentrated force of the magnetic force is:

(9) $$F=\sum_{i=1}^{n}F_{i}$$

**Figure 3:**
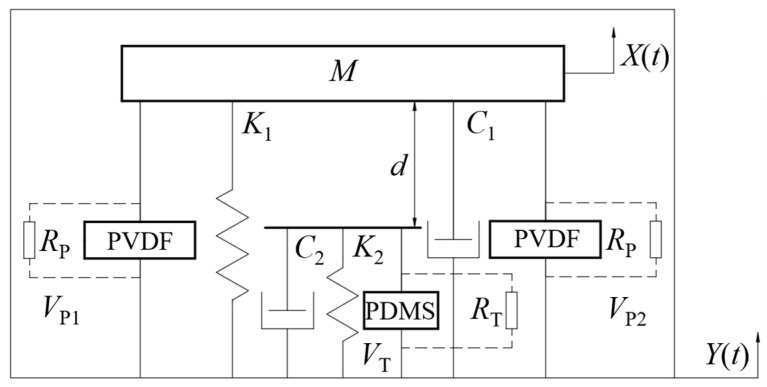
Kinetic model schematic representation.

As a result, the rotating energy harvester is subjected to an external excitation *F*(t). The rotational energy harvester can be reduced to a spring-damped model, and a kinetic model schematic representation of the system is shown in Figure 3. *M*, *K*_1_, *C*_1_ is the equivalent mass, equivalent stiffness and equivalent damping of the Charpy beam of the piezoelectric unit, *R*_P_ is the load resistance of the piezoelectric film, *V*_P_(*t*) is the output voltage of the piezoelectric film, *X*(*t*) is the moving displacement of the Charpy beam, *Y*(*t*) is the external excitation of the Charpy beam, *d* is the spacing between the triboelectric units when the composite energy harvester is stationary, *K*_2_ and *C*_2_ are the collision stiffness and additional damping at the time of collision respectively, *R*_T_ is the load resistance of the triboelectric unit, and *V*_T_(*t*) is the output voltage of the triboelectric unit.

Based on the fundamental principles of dynamics, the motion control equations of the system can be established as follows:

(10) $$M\frac{d^{2}X(t)}{dt^{2}}+C_{1}\frac{dX(t)}{d(t)}+K_{1}X(t)+F_{P}+F_{S}=M\frac{d^{2}Y(t)}{dt}$$

During the triboelectric unit contact:

(11) $$E_{T}=-\frac{Q_{T}(t)}{S\varepsilon_{0}\varepsilon_{T}}$$

(12) $$E_{\mathrm{Air}}=-\frac{\frac{Q_{T}(t)}{S}+\sigma (t)}{\varepsilon_{0}}$$

where $Q_{T}(t)$ is the amount of charge transferred; S is the contact area of the positive and negative polarity dielectric materials; $\varepsilon_{0}$ is the vacuum dielectric constant; $\varepsilon_{T}$ is the relative permittivity of the PDMS negative triboelectric material; σ(t) is the surface charge density of the PDMS negative triboelectric material.

If the distance between the triboelectric electrodes is *x*(*t*), then the potential difference in the triboelectric unit:

(13) $$V_{T}(t)=E_{T}d_{T}+E_{\mathrm{Air}}x(t)=-\frac{Q_{T}(t)}{S\varepsilon_{0}}[\frac{d_{T}}{\varepsilon_{T}}+x(t)]+\frac{\sigma x(t)}{\varepsilon_{0}}$$

where $d_{T}$ is the thickness of the negative polarity triboelectric layer material.

When the resistor $R_{T}$ is connected, the dynamics equation for the electromechanical coupling of the triboelectric element is:

(14) $$R_{T}\frac{dQ_{T}(t)}{dt}=-\frac{Q_{T}(t)}{S\varepsilon_{0}}[\frac{d_{T}}{\varepsilon_{T}}+x(t)]+\frac{\sigma x(t)}{\varepsilon_{0}}$$

Post-coupling stress $F_{P}$:

(15) $$F_{P}=-\theta_{V}V_{P}(t)$$

where $\theta_{V}$ is the post-coupling coefficient.

The force *F*_S_ generated by the contact separation of the triboelectric unit is:

(16) $$F_{S}=\left\{\begin{array}{cc} 0, & X(t)>-d\\ C_{2}\frac{dX(t)}{dt}+K_{2}[X(t)+d], & X(t)\leq -d \end{array}\right.$$

The overall electromechanical coupling dynamics equation is:

(17) $$\left\{\begin{array}{l} M\frac{d^{2}X(t)}{dt^{2}}+C_{1}\frac{dX(t)}{dt}+K_{1}X(t)-\theta_{V}V_{P}(t)+F_{S}=F(t)\\ \frac{V_{P}(t)}{R_{P}}+C_{1}\overset{\cdot }{V_{P}}(t)-\theta_{V}\frac{dX(t)}{d(t)}=0\\ R_{T}\frac{dQ_{T}(t)}{dt}=-\frac{Q_{T}(t)}{S\varepsilon_{0}}[\frac{d_{T}}{\varepsilon_{T}}+x(t)]+\frac{\sigma x(t)}{\varepsilon_{0}} \end{array}\right.$$

where x(t) is the distance from the surface of the triboelectric film.

## 3. Simulation Analysis of Composite Rotary Vibration Energy Harvester

### 3.1. Structural Analysis of Energy Harvester

**Figure 4:**
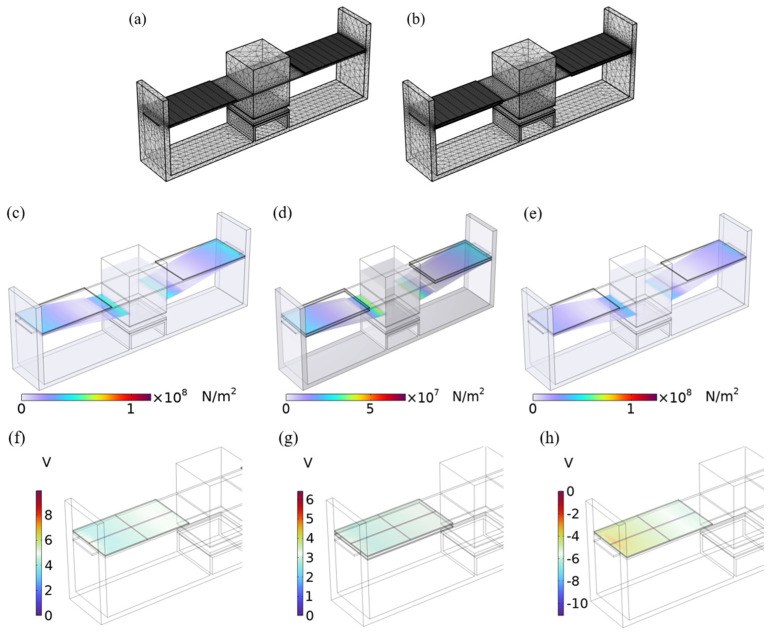
Stress distribution and output voltage diagram of the two structures under fixed load: (**a**) Single–crystal structure; (**b**) twin structure; (**c**) stress of single–crystal PVDF; (**d**) twin PVDF stress; (**e**) stress of single–crystal PZT–4; (**f**) monocrystalline PVDF voltage; (**g**) dual PVDF voltage; (**h**) monocrystalline PZT–4 voltage.

The structure selection of the energy harvester plays a key role in the design of self-powered systems, directly influencing the output voltage performance. Generally, piezoelectric beams fall into two categories: single-crystal and double-crystal configurations. COMSOL6.1 software was utilized to model and mesh these two structures, aiming to investigate their impact on the output voltage; the finite element models are illustrated in Figure 4a,b. The bottom surface, side of the support seat and both sides of the piezoelectric material were applied to the supporting constraints for simulation analysis. Table 2 shows the dimensions of the basic module of the energy harvester. The thickness of the Al plate and PVDF piezoelectric film (Zhimikang Technology Co., Ltd., Shenzhen, China) is 0.1 mm, the thickness of the PMDS triboelectric film (Zhongke Materials Co., Ltd., Shenzhen, China) is 0.5 mm, and the spacing between the friction layers of the triboelectric unit is set to 0.5 mm. The material parameters of the energy harvester can be obtained from Table 3.

A fixed load of 3 N is applied to the magnetic pole of the upper mass, and the stress distribution of the Charpy energy harvester is shown in Figure 4c–e, and the corresponding open-circuit voltages are shown in Figure 4f–h. The results show that the stress distribution of the energy harvester is more uniform when the single-crystal simply supported beam structure is adopted, and the output voltage of a single piezoelectric unit reaches 9.92 V, which is higher than that of the dual-crystal structure. Although the dual-crystal structure has two piezoelectric units, the output voltage of a single unit is low, and the impedance of the upper and lower piezoelectric units is high when they are connected in series, resulting in a large energy loss.

**Figure 5:**
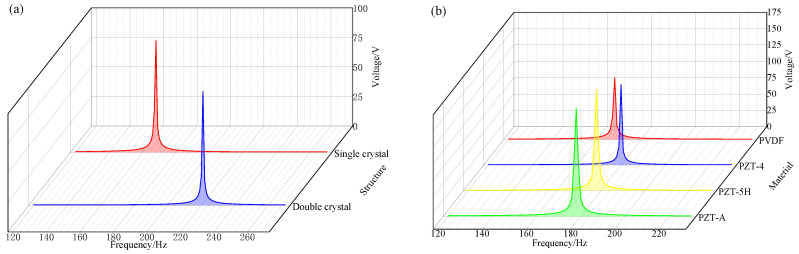
Output voltage and vibration frequency response curves for different structures and materials: (**a**) Piezoelectric output voltage and vibration frequency response curves of the two structures; (**b**) piezoelectric output voltage and vibration frequency response curves of different materials.

Under the condition that the other conditions of the energy harvester remain unchanged, an acceleration excitation of 1 m/s^2^ is applied to the Charpé beam, and the relationship between the single and double crystal structure and the natural frequency and output voltage is obtained through frequency domain analysis, as shown in Figure 5a. The results show that the natural frequency of the monocrystalline structure is low, but its peak voltage is similar to that of the dual-crystal structure, which is about 90 V. In addition, the single-crystal structure has the advantages of simple structure and convenient fabrication. Combined with the results of the above-mentioned fixed load analysis, the single-crystal cantilever beam structure was finally selected.

The specific parameters of commonly used piezoelectric materials (e.g., PZT and PVDF) are shown in Table 4. Under unchanged conditions, the relationship between the first-order natural frequency and the output voltage from frequency domain analysis is given in Figure 5b. The results show that the first-order natural frequency of PVDF piezoelectric material is 167 Hz, which is lower in frequency and easier to excite vibration. As shown in Figure 4c,e,f,h, although the output voltage of the PVDF material is lower, the maximum stress at the connection is also the lowest. Although the output voltage peak of PVDF is 95 V, which is lower than that of PZT-5H, PZT-4, and PZT-5A, it can be seen from Table 2 that PZT-5H, PZT-4, and PZT-5A are more brittle and have a higher hardness, and are prone to fatigue damage, while PVDF has excellent flexibility and can still maintain good performance under high-frequency vibration. Therefore, PVDF material is selected as the piezoelectric material.

**Figure 6:**
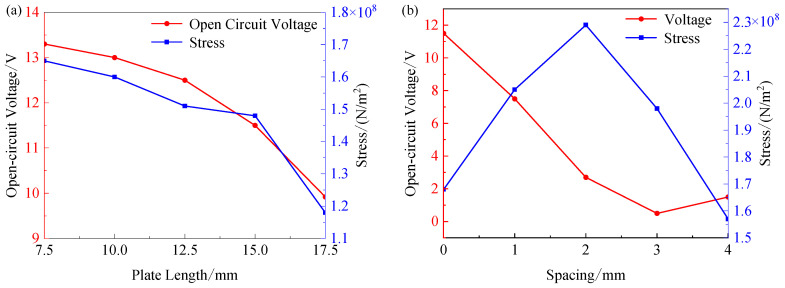
Plate length and position analysis: (**a**) Maximum stress and output voltage versus plate length; (**b**) maximum stress and output voltage versus plate fixed end spacing.

By analyzing the variation law of the output open-circuit voltage and the maximum stress of the energy harvester under fixed load, the optimal length of the piezo electrode plate and the optimal distance between the plate and the side plate of the base can be determined. The magnetic force on the energy harvester is simulated by setting an external load force of 3 N, and the maximum stress and output voltage as a function of plate length and fixed end spacing are shown in Figure 6. As can be seen from Figure 6a, the open-circuit voltage of the piezoelectric unit of the energy harvester gradually decreases as the plate length increases, while the maximum stress decreases. Combined with the results of the analysis of output voltage and stress, the final plate length was selected to be 12.5 mm. Figure 6b reveals that increasing the fixed end spacing causes the output voltage of the piezoelectric unit to first decrease and then increase, in contrast to the stress, which first increases and then decreases. Therefore, the energy harvester’s output voltage is optimal when the spacing is zero.

**Figure 7:**
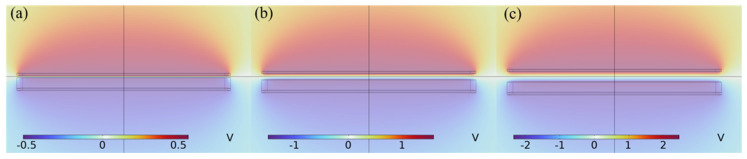
The connection between the distance of friction layers and the open–circuit potential: (**a**) 0.1 m; (**b**) 0.3 mm; (**c**) 0.5 mm.

A contact-separation triboelectric nanogenerator was set between the lower mass and the support base boss as a triboelectric unit. Combined with the structural size analysis of the energy harvester, the spacing of the static triboelectric units of the design system is 0.5 mm, and when the spacing of the triboelectric electrodes returns to 0.5 mm after contact, the friction voltage reaches the saturation value, and the simulation results of the output voltage of the triboelectric electrode with the spacing of the triboelectric electrodes are shown in Figure 7a–c. As can be seen from Figure 7a–c, when the spacing does not exceed 0.5 mm, the friction voltage gradually increases as the spacing of the friction electrodes increases. When the spacing reaches 0.5 mm, the open-circuit voltage reaches the saturation value of 4.81 V.

### 3.2. Simulation Analysis of Magnetic Module

**Figure 8:**
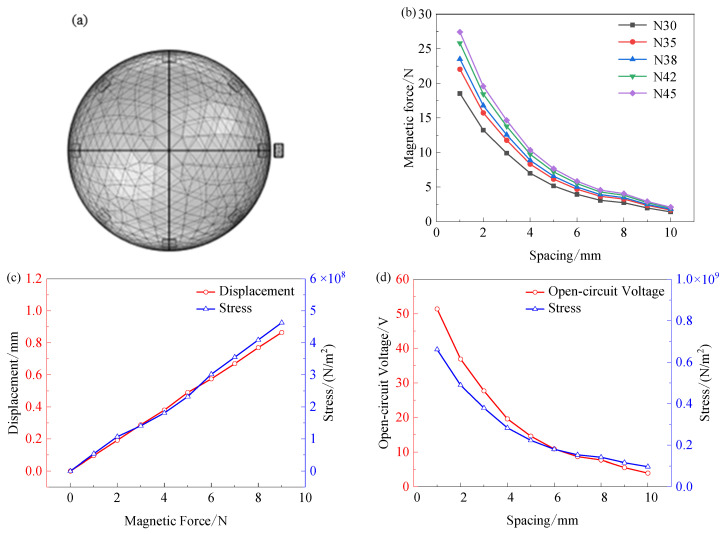
Simulation analysis of magnetic drive module: (**a**) Meshing; (**b**) the magnetic force of different materials varies with spacing; (**c**) curves of displacement and stress as a function of electromagnetic force; (**d**) the curve of open-circuit voltage and stress with spacing.

The main factors affecting the open-circuit voltage of the magnetic module are materials and structures. In the COMSOL software, the rotation domain was established with the Z-axis as the center, the rotating magnetic poles were uniformly distributed on a circle with an outer diameter of 124 mm to simulate the movement of the magnetic poles on the outer ring expansion ring during bearing operation, and the fixed magnetic poles were set in the X-axis direction to simulate the fixed magnetic poles on the energy harvester, and the rotational speed was set to 600 r/min, and the rotating machinery-magnetic module was studied, as shown in Figure 8a. NdFeB series magnets are ideal material choices due to their long life, strong magnetism, and stable magnetism under high-frequency vibration. NdFeB magnets with different compositions: N30, N35, N38, N42, and N45 magnets were used for analysis. Figure 8b shows that the electromagnetic force generated by the N45 magnet in the pole direction is always better than that of other magnets under different spacing conditions, and the performance is better.

Combined with the above analysis, the N45 magnet was selected, and the influence of the distance between the magnets on the output voltage of the energy harvester was further analyzed. The displacement deformation of the energy harvester under the action of the electromagnetic force is shown in Figure 8c. To ensure the output voltage amplitude of the composite energy harvester remains stable, the maximum reverse displacement at the mass end should be controlled at about 0.5 mm. At this time, the energy harvester is subjected to an electromagnetic force of about 5 N, and the maximum stress of the energy harvester is about 2.1 × 10^8^ N/m^2^. To ensure the stable operation of the energy harvester, the maximum stress should be around 2.1 × 10^8^ N/m^2^ when the optimal spacing is reached. Figure 8d shows the open-circuit voltage and stress decrease as the spacing decreases. When the spacing is 6 mm, the maximum stress is about 2.1 × 10^8^ N/m^2^ and the open-circuit voltage reaches the optimal value. Therefore, the spacing between magnets is 6 mm.

**Figure 9:**
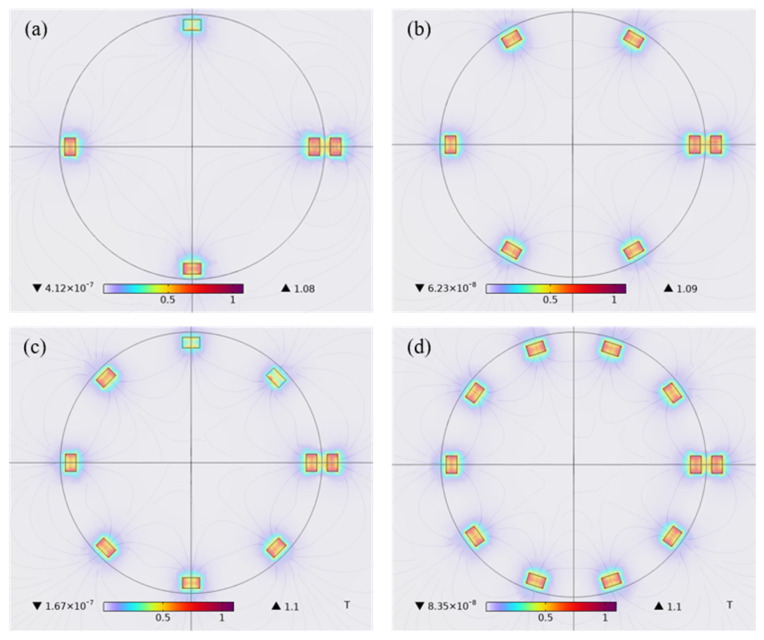
Magnetic flux density diagram for different rotor numbers: (**a**) 4–pole; (**b**) 6–pole; (**c**) 8–pole; (**d**) 10–pole.

The magnetic flux density B directly reflects the effective strength of the magnetic field, which determines the ability of the magnetic field to act on the electric current, the magnetic material and the moving charge. By studying the magnetic flux density strength of the magnetic field at different rotors, the selection of the rotor in the outer ring of the energy harvesting system was analyzed. Keeping other conditions unchanged, the magnetic flux density performance of 4, 6, 8, and 10 rotors at steady state is compared through finite element simulation, as shown in Figure 9a–d. When the number of rotors is eight or 10, the magnetic flux density is the largest, which can reach 1.1 T.

**Figure 10:**
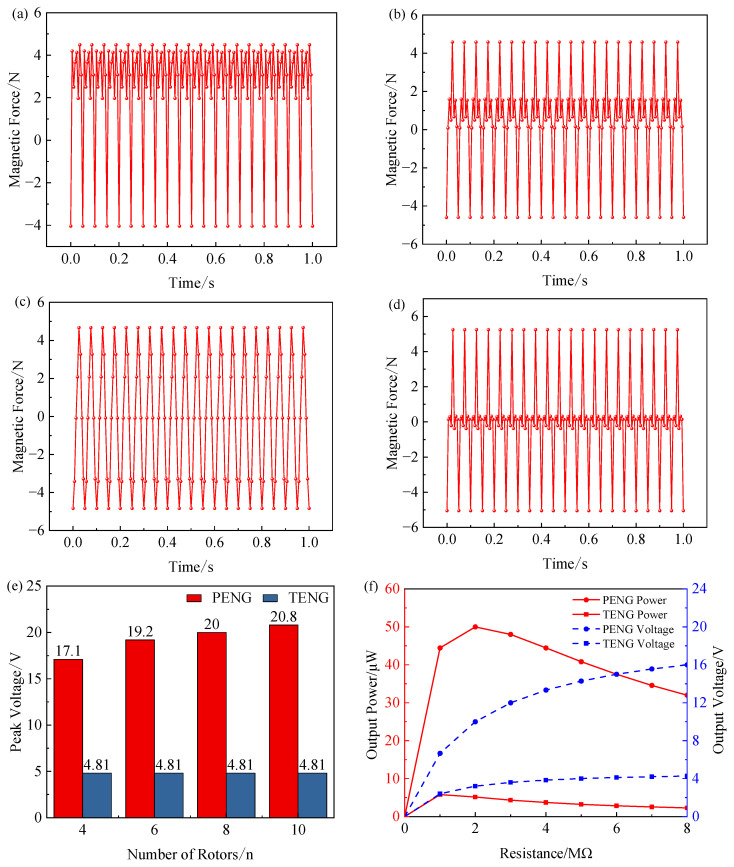
Output characteristic curve: (**a**) 4–rotor magnetic output; (**b**) 6–rotor magnetic output; (**c**) 8–rotor magnetic output; (**d**) 10–rotor magnetic output. (**e**) The correlation between rotor count and output voltage. (**f**) Load vs. output power curve.

By directly determining the bending amplitude of the Charpy piezoelectric unit and the contact-separation amplitude of the triboelectric unit, the electromagnetic force magnitude plays a key role in shaping the output voltage characteristics of the composite energy harvester. Keeping other conditions unchanged, the finite element simulation method is used to systematically study the variation law of the electromagnetic force of the energy harvester when the number of magnetic pole rotors is four, six, eight and 10, and then the correlation between the output voltage characteristics and the number of rotors is analyzed, and finally the influence of the number of rotors on the output performance of the composite energy harvester is revealed. When the pole speed is 300 r/min, the electromagnetic force generated by different rotor numbers is shown in Figure 10a–d, and the peak piezoelectric voltage and peak triboelectric voltage under different rotors are shown in Figure 10e. The analysis of the results of Figure 10 shows that the electromagnetic force on the energy harvester changes periodically, and when there are four rotors, the spacing between different rotors is larger, the interaction is small, and the peak electromagnetic force generated is 4.1 N, and the peak piezoelectric voltage and triboelectric voltage generated at this time are 17.1 V and 4.81 V. In the case of six rotors, eight rotors, and 10 rotors, the distance between the magnetic poles is close, and the peak electromagnetic force and voltage generated are similar. Figure 10 shows that the electromagnetic force output is more stable at eight rotors, and the time of equipotential output is shorter, which can effectively reduce the output waveform distortion and improve the overall energy conversion efficiency. Therefore, an eight-pole rotor structure was chosen. In this case, the piezoelectric and triboelectric units exhibit peak output voltages of 20 V and 4.81 V, respectively. The dependence of the maximum output power on load resistance is presented in Figure 10f. Specifically, at an external load of 2 MΩ, the piezoelectric unit delivers 50 μW, whereas at 1 MΩ, the triboelectric unit provides 5.8 μW.

## 4. Design of Hybrid Energy Harvesting Circuits

**Figure 11:**
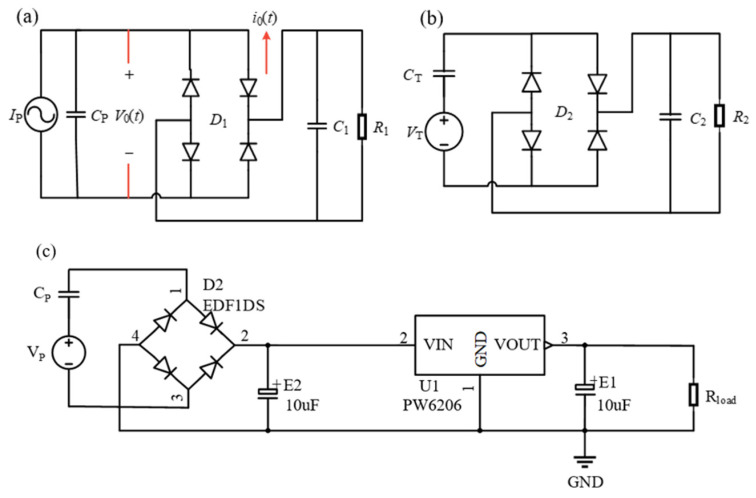

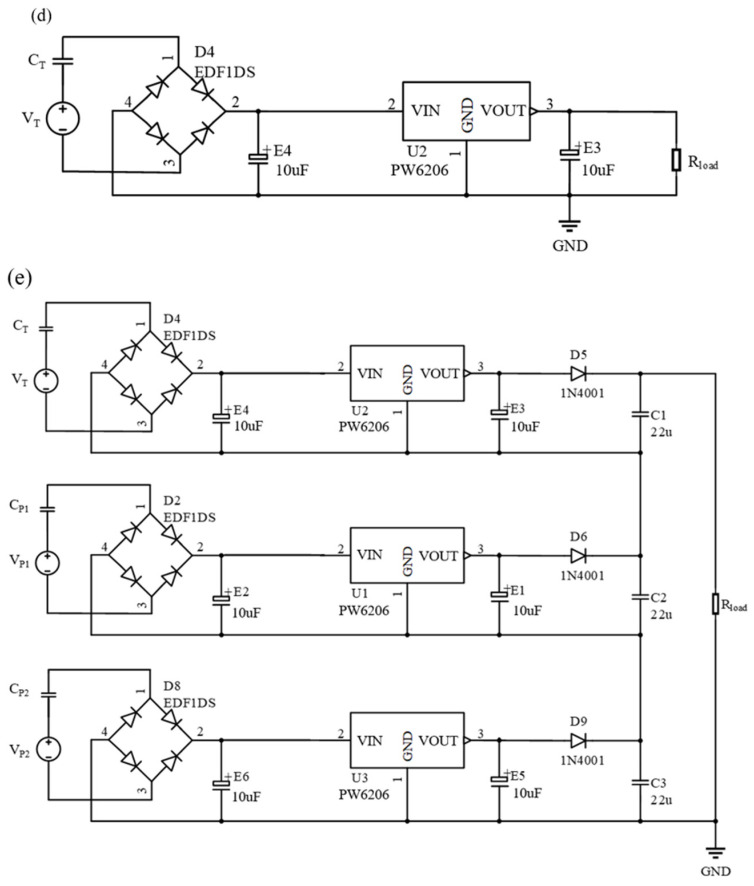
(**a**) Current-source model of a standard energy harvesting circuit. (**b**) Voltage-source model of a standard energy harvesting circuit. (**c**) Energy harvesting circuit for a piezoelectric unit. (**d**) Energy harvesting circuit for a triboelectric unit. (**e**) Energy harvesting circuit for a hybrid energy harvester.

The hybrid energy harvester within the smart bearing provides excitation to the system through periodic magnetic forces; specifically, when this periodic magnetic force takes the form of a sinusoidal acceleration excitation, the hybrid energy harvester outputs a corresponding sinusoidal voltage. The equivalent circuit model of the piezoelectric unit can be constructed using a current source in parallel with a capacitor; the standard current-source model for such an energy harvesting circuit is illustrated in Figure 11a, while the voltage-source model is shown in Figure 11b. Under the excitation of rotating magnetic forces, the hybrid energy harvester designed in this paper outputs alternating current (AC), which is generally unsuitable for meeting the operational requirements of conventional power-consuming devices. Therefore, building upon the standard energy harvesting circuit, this section incorporates the PW6206 linear voltage regulator chip. This integration enables the filtering capacitor to additionally serve the function of charge storage, thereby achieving the objective of outputting a stable direct current (DC) voltage. The PW6206 linear voltage regulator chip is capable of converting a higher, unstable input voltage (ranging from 4 V to 40 V) into a fixed, stable, and lower output voltage (ranging from 1.8 V to 5.7 V), thereby effectively providing power to conventional power-consuming devices. Based on the fundamental principles of Thevenin’s and Norton’s theorems, a parallel combination of a current source and a capacitor can be equivalently transformed into a series configuration comprising a voltage source and a capacitor. Consequently, a piezoelectric unit can be modeled as an equivalent circuit consisting of a voltage source connected in series with a capacitor. Simulation analysis reveals that the piezoelectric unit generates an AC output voltage with a peak amplitude of 20 V; therefore, utilizing the PW6206 linear voltage regulator IC in conjunction with a standard rectifier bridge energy management circuit, a piezoelectric energy harvesting circuit—as illustrated in Figure 11c—was constructed. During operation, the sinusoidal AC signal VP generated by the piezoelectric unit is converted into pulsating DC power by the rectifier bridge D2. This pulsating DC is then subjected to low-pass filtering by the aluminum electrolytic capacitor E2, transforming it into a relatively smooth DC voltage which is temporarily stored within the capacitor. Subsequently, this electrical energy is fed from the input interface VIN into the PW6206 linear voltage regulator IC (U1); after internal regulation, the chip outputs a lower, stable voltage that is stored in the aluminum electrolytic capacitor E1, thereby supplying power to the external load Rload. Generally, the equivalent circuit model of a triboelectric unit can be represented as a series combination of a voltage source and a capacitor; consequently, the triboelectric unit outputs a periodically varying voltage signal. Therefore, the design process for the triboelectric energy harvesting circuit mirrors that of the piezoelectric energy harvesting circuit, as illustrated in the circuit diagram in Figure 11d. In the final hybrid energy harvesting circuit, the piezoelectric and triboelectric units are connected to separate rectification and voltage regulation modules based on the PW6206 chip (Pingxinwei Semiconductor Technology Co., Ltd., Wuxi, China). Each rectification and regulation module is connected via an external 1N4001 diode (Mengke Electronics Co., Ltd., Shenzhen, China) in parallel with a dedicated energy storage capacitor; ultimately, the storage capacitors from these individual branches are connected in series to power the load. The overall logic diagram for the energy harvesting circuit is depicted in Figure 11e.

## 5. Output Characteristics Test of Magnetic Coupling Piezoelectric–Triboelectric Composite Rotary Energy Harvester

**Figure 12:**
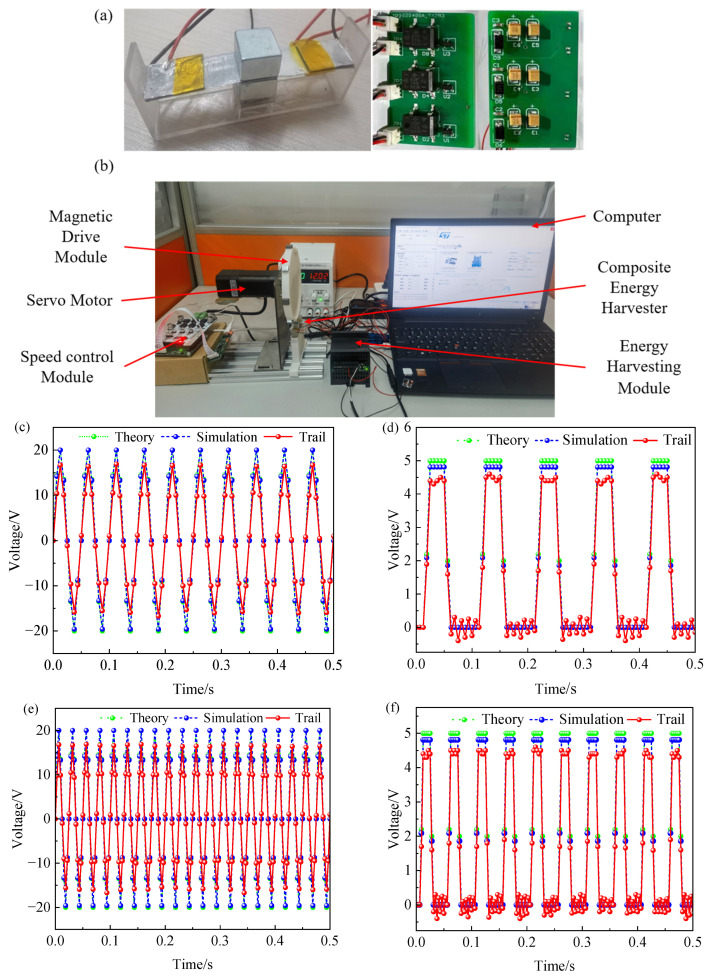

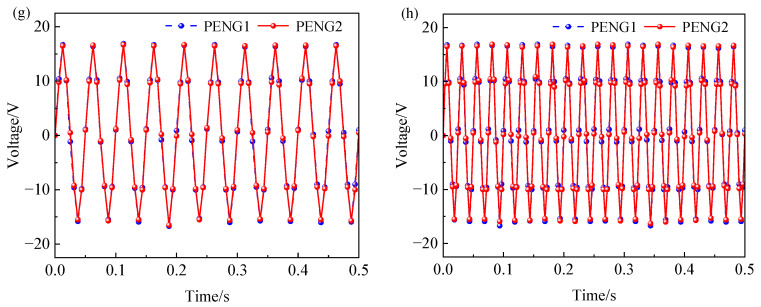
The energy harvester outputs piezoelectric voltage and triboelectric voltage at different speeds: (**a**) Energy harvester and energy harvesting circuit prototype; (**b**) schematic diagram of the test platform system; (**c**) piezoelectric unit’s open-circuit voltage at 300 r/min; (**d**) triboelectric unit’s open-circuit voltage at 300 r/min; (**e**) piezoelectric unit’s open-circuit voltage at 600 r/min; (**f**) triboelectric unit’s open-circuit voltage at 600 r/min; (**g**) comparison of piezoelectric unit output voltages at 300 r/min; (**h**) comparison of piezoelectric unit output voltages at 600 r/min.

In order to verify the output characteristics of the magnetic coupling composite rotary energy harvester, a prototype of the composite energy harvester was designed and fabricated, and a rotary test platform was built to simulate the effect of the rotating magnetic force on the composite energy harvester under the condition of low speed and light load of a 6220 deep groove ball bearing. The main components of the test platform include a speed-regulating motor, a speed-regulating controller, an energy harvesting module, a data acquisition card, a computer, etc. A magnetic pole fixing ring and a composite energy harvester together form the energy harvesting module. Among them, the outer diameter of the magnetic pole fixing ring is 124 mm, and 8 S/N alternating magnetic poles (Qiantang Magnetic & Electric Co., Ltd., Hangzhou, China) are evenly distributed on the surface, with a minimum spacing between the magnetic poles of 6 mm; the composite energy harvester is fixed just below the magnetic pole retaining ring. The configuration scheme of the module is consistent with the rotation radius of the inner ring expansion ring of the intelligent bearing and the minimum spacing between the magnetic poles, and the prototype and test platform are shown in Figure 12a,b. Through the FL42SVA03-6 speed-regulating motor (Siemens CNC Co., Ltd., Nanjing, China), the composite energy harvester is subjected to the action of rotating magnetic force and generates an electrical signal under the action of magnetic force, which is recorded and processed by the DAQ1402 data acquisition card (Conway Technology Co., Ltd., Shenzhen, China) and stored on the computer.

In the test, other conditions are set unchanged, and the speed of the speed-regulating motor is set to 300 r/min and 600 r/min, and the output voltage change is recorded. In the initial state, there is no contact between the triboelectric units, and no frictional voltage is generated. With the contact separation between the triboelectric electrodes, the triboelectric unit starts working. Figure 12c–f presents the open-circuit voltages of the single-sided piezoelectric unit and the triboelectric unit at rotational speeds of 300 r/min and 600 r/min. Table 5 summarizes several parameters of the theoretical model. It can be seen from the figure that the rotation velocity has no significant effect on the output voltage amplitude of the composite energy harvester, but it has a significant positive correlation with the output voltage frequency. Compared with the piezoelectric–triboelectric composite vibration energy harvester [29], which is excited by the vibration of the bearing itself, the peak voltage of the piezoelectric unit is about 3 V, and the peak voltage of the triboelectric unit is about 0.9 V, and the peak voltage of the piezoelectric unit can reach about 16.6 V and the peak voltage of the triboelectric unit under the normal operation of the composite energy harvester based on magnetic coupling is 4.4 V. At rotational speeds of 300 r/min and 600 r/min, the voltages generated by the two piezoelectric units are compared in Figure 12g,h; as evident from the figures, there is no phase lag or lead between the output voltages of the two piezoelectric units.

**Figure 13:**
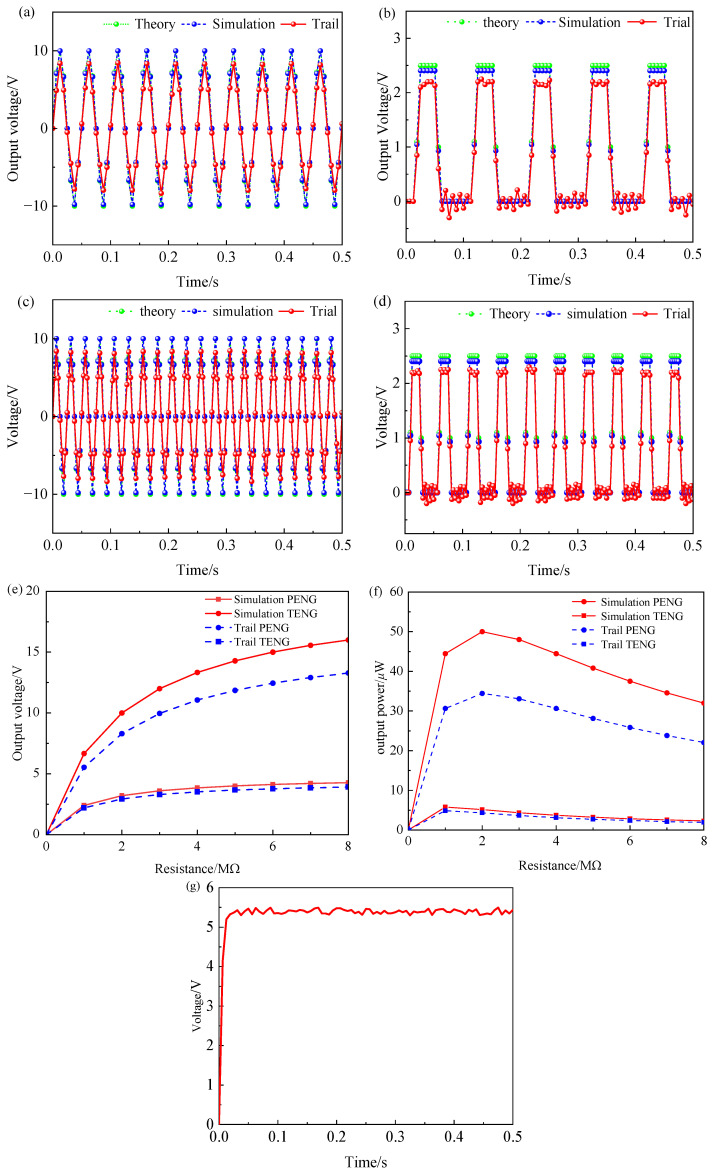
Load output characteristics analysis: (**a**) Output voltage of the piezoelectric unit connected to an external load at 300 r/min; (**b**) output voltage of the triboelectric unit connected to an external load at 300 r/min; (**c**) output voltage of the piezoelectric unit connected to an external load at 600 r/min; (**d**) output voltage of triboelectric unit connected to external load at 600 r/min; (**e**) simulated and experimental output voltage; (**f**) simulated and experimental output power; (**g**) energy harvesting circuit output voltage.

The piezoelectric and triboelectric units are respectively connected to loads of 2 MΩ and 1 MΩ. Figure 13a–d depicts the resulting output voltages at 300 r/min and 600 r/min. Under these conditions, the single-sided piezoelectric unit generates a peak voltage of approximately 8.5 V, and the triboelectric unit produces 2.2 V. The relationship between output power/voltage and external load resistance is presented in Figure 13e,f. The maximum attainable output power is 35.28 μW for the single piezoelectric unit and 4.84 μW for the triboelectric unit. When the hybrid energy harvester is connected to the hybrid energy harvesting circuit, the output voltage—following rectification and voltage regulation by the circuit—is as shown in Figure 13g. As can be observed from Figure 13g, the output voltage of the energy harvesting circuit rises to approximately 5.4 V within about 12 ms and subsequently stabilizes; this meets the conditions required for the stable operation of typical sensors.

## 6. Conclusions

In this paper, a piezoelectric–triboelectric composite energy harvester driven by a rotating magnetic force is designed for the self-power supply requirements of intelligent bearings, which is summarized as follows:

(1) It adopts an outward extension intelligent bearing structure, which is composed of a 6220 deep groove ball bearing, outer ring collar, inner ring expansion ring, magnetic pole and composite energy harvester. The energy harvester vibrates through magnetic coupling and converts the vibration energy of the rotational motion into electrical energy to power the intelligent bearing. The energy harvester adopts the piezoelectric–triboelectric composite method to collect vibration energy more effectively.
(2) Through the simulation analysis of COMSOL software, the influence of different structural sizes and magnetic poles on the output voltage was analyzed, and the structural dimensions of the extended rotary module and the composite energy harvester were determined. The structure of the composite energy harvester is a single-crystal structure, with a piezoelectric material PVDF, the piezoelectric material length is 12.5 mm, with eight magnetic poles, and the magnetic pole spacing is 6 mm. Through simulation analysis, it can be seen that the open-circuit voltage of the piezoelectric unit can reach 20 V, and the open-circuit voltage of the triboelectric unit can reach 4.81 V.
(3) The electromechanical coupling dynamic model of the composite energy harvesting system was validated via an open-circuit voltage test scheme. Experimental results confirmed its accuracy alongside simulation analysis. The consistency between theoretical, simulation, and experimental outcomes demonstrates the model’s excellent characterization capability. Test results show that under normal magnetic coupling-based operation, the open-circuit peak voltage is about 16.6 V for the single-sided piezoelectric unit and approximately 4.4 V for the triboelectric unit. The output power of a single piezoelectric unit can reach up to 35.28 μW with an external load of 2 MΩ and up to 4.84 μW with a load of 1 MΩ. The output voltage of the energy harvesting circuit rises to approximately 5.4 V within about 12 ms and subsequently stabilizes; this meets the conditions required for the stable operation of typical sensors.

## Figures and Tables

**Table 1:** Overall structural geometric parameters and materials.

Component	Geometric Parameters	Material
6220 Deep Groove Ball Bearing	180 mm outside, 100 mm inside diameter	Bearing Steel
Outer Ring Sleeve	220 mm outside, 160 mm inside diameter	alloy steel
Inner Ring Extension Ring	124 mm outside, 110 mm inside diameter	alloy steel
Magnetic Pole	10 mm in length/width, 6 mm in height	NdFeB

**Table 2:** Structural parameters of the energy harvester.

Structure	Support Seat Base Plate/mm^3^	Support Seat Side Panels/mm^3^	Mass/mm^3^	Boss Side Plates/mm^3^
size	56.6 × 10 × 1	19 × 10 × 1.3	10 × 10 × 6	10 × 4 × 0.5

**Table 3:** Material parameters.

Parameter	Young’s Modulus/GPa	Poisson’s Ratio	Density ρ/(kg/m^3^)	Coupling Coefficient d_31_/(pC/N)	Dielectric Constant *ε*_33_
Al	70	0.35	2700	/	/
PMMA	3	0.32	1150	/	/
PVDF	2.5	0.3	1780	21	13.5
NdFeB	160	0.28	7400	/	/
PDMS	0.75	0.49	970	/	27

**Table 4:** Piezoelectric material parameter table.

Material Parameters	PZT-4	PZT-5H	PZT-5A	PVDF
$\xi_{33}^{S}$	635	1470	910	5–13
$\xi_{33}^{T}$	1300	3400	1200	7.6
$Q_{m}$	500	65	400	3–10
$\Theta_{33}$	0.7	0.75	0.49	0.19
$s_{11}^{E}$/(p·Pa^−1^)	12.3	16.4	8.6	365
$s_{33}^{E}$/(p·Pa^−1^)	15.5	20.8	9.1	472

Note: $\xi_{33}^{S}$ is the tensile stress constant, $\xi_{33}^{T}$ is the compressive stress constant, $Q_{m}$ is the mechanical quality factor, $\Theta_{33}$ is the electromechanical coupling coefficient, $s_{33}^{E}$ and $s_{11}^{E}$ is the compliance coefficient.

**Table 5:** Some parameters of the theoretical model.

$\varepsilon_{0}$	$\varepsilon_{T}$	$\sigma_{T}$ (C/m^2^)	$d_{T}$ (mm)	S (mm^2^)
1	2.75	1 × 10^−7^	0.5	100

## Data Availability

Research data will be available upon reasonable request.
